# Genomic and Transcriptomic Profiling of *Bacillus cereus* in Milk: Insights into the Sweet Curdling Defect

**DOI:** 10.3390/foods14050780

**Published:** 2025-02-25

**Authors:** Maria Kyritsi, George Tsiolas, Antiopi Tsoureki, Vasiliki Schoretsaniti, Maria Gougouli, Sofia Michailidou, Anagnostis Argiriou

**Affiliations:** 1 Institute of Applied Biosciences, Centre for Research and Technology Hellas, 57001 Thessaloniki, Greece; kyritsimaria@certh.gr (M.K.); gtsiolas@reframe.food (G.T.); adatsoureki@certh.gr (A.T.); michailidouso@certh.gr (S.M.); 2 Reframe Food Astiki Mi Kerdoskopiki Etairia, 57001 Thessaloniki, Greece; 3 MEVGAL S.A., 57100 Thessaloniki, Greece; baswsxor@gmail.com (V.S.); mgougouli@mevgal.gr (M.G.); 4 Department of Food Science and Nutrition, University of the Aegean, 81400 Myrina, Greece

**Keywords:** sweet curdling, milk spoilage, *Bacillus cereus*, virulence, whole genome sequencing (WGS), transcriptome sequencing, differential gene expression analysis (DGE)

## Abstract

*Bacillus cereus sensu lato* (*B. cereus s.l.*) are significant spoilage and pathogenic microorganisms found in various foodstuffs. They are responsible for defects like sweet curdling in milk, which impacts dairy product storage and distribution. Nevertheless, the genetic mechanisms underlying *B. cereus*-induced sweet curdling remain poorly characterized. In this study, we investigated the genetic and functional basis underlying this phenomenon through whole genome sequencing of the newly isolated *B. cereus* strain BC46 and transcriptome sequencing at two phases of its growth in milk. Hybrid assembly of Illumina and Nanopore reads resulted in a 5.6 Mb genome with 35.1% GC content, classifying BC46 as *B. cereus sensu stricto* (*B. cereus s.s.*) within the *panC* group IV. Several virulence factors, antimicrobial resistance genes, and cold shock proteins were identified in the genome. A distinct functional profile of BC46 was observed before and after the development of sweet curdling in milk. Genes associated with sporulation, toxin production, hydrolysis, and proteolysis were upregulated in sweet-curdled samples. Our findings highlight potential gene targets that may play an important role in the BC46-induced sweet curdling in milk, enhancing our understanding of its molecular basis and supporting the development of new genetic approaches for early spoilage detection.

## 1. Introduction

*Bacillus cereus sensu lato* (*B. cereus s.l.*), also known as the *Bacillus cereus* group, is a phylogenetically complex group of spore-forming, facultative anaerobic bacteria. The group comprises the well-known species *B. cereus sensu stricto* (*s.s.*), *B. anthracis*, and *B. thuringiensis*, which have major medical and economic importance, as well as several other species with closely related phylogeny [1]. *B. cereus s.s.* is the most common species of the group implicated in food poisoning cases [2]. In addition to its pathogenic effects, commonly causing diarrheal and emetic food poisoning [3], it also contributes to milk spoilage, posing significant challenges to production processes and affecting the quality of dairy products [4].

*B. cereus s.l.* are among the dominant bacteria in dairy processing environments causing the premature spoilage of products [5]. These bacteria primarily originate from environmental sources, as they are abundant in soil, raw materials, and milking equipment associated with dairy farming [6]. Despite stringent hygiene protocols implemented during dairy processing, post-processing contamination (PPC) and fluctuations in cold chain maintenance facilitate bacterial proliferation along the production line [7,8]. The formation of heat-resistant spores and psychrotrophic growth during storage pose significant restrictions in the shelf-life extension of dairy products. These bacteria form resilient endospores that are induced by and can survive pasteurization [9], including ultra-high temperature (UHT) processes (135–150 °C) [10]. Other key spoilage mechanisms include the formation of biofilms that act as strong shields for spores and vegetative cells and the production of enzymes that lead to protein breakdown and fat hydrolysis in milk [11,12]. The significant contamination of raw milk by *B. cereus s.l.* has been documented globally, with recent research indicating prevalence rates ranging from 33.0 to 37.5% [13,14] to 47.0 to 100.0% [15,16]. Additionally, they have been detected at 7.5–25.0% in UHT-treated milk samples [12,14,17]. Notably, even moderate variations in refrigeration temperatures significantly affect *B. cereus* levels in pasteurized milk, with an increase of 12.6% when the temperature rises from 4 °C to 7 °C, and a 40.0% increase when it rises from 4 °C to 10 °C [18]. The persistence of these bacteria throughout the dairy production chain highlights the need for targeted interventions to mitigate contamination in the dairy processing environment.

*B. cereus s.l.* produce heat-stable, extra-, or intra-cellular enzymes, such as proteases, lipases, and phospholipases, that lead to undesirable sensory profiles in milk and its derivatives, such as rancidity, butyric flavors, and soapy textures [19,20,21]. Proteases exhibit diverse functional activities that often result in coagulation and bitterness, contributing to defects such as the “sweet curdling” of fluid milk [22,23]. This defect is characterized by the development of a curd with creamy texture, without a significant pH decrease—rather than the sour curd associated with lactic acid fermentation—resulting in an undesirable bitter flavor. This phenomenon typically occurs late in milk’s shelf life, particularly at high bacterial populations exceeding 10^6^ CFU/mL, primarily caused by spore-forming *Bacillus* species, such as *B. cereus*, *B. weihenstephanensis*, and others [24,25]. In refrigerated pasteurized milk, the initial indication of this defect is the formation of a curd layer at the bottom of the container but without the typical sour flavor or odor commonly associated with milk spoilage [26]. The absence of distinctive sensory indicators complicates early detection of sweet curdling, often allowing defective batches to proceed through storage and distribution channels, eventually leading to increased product waste and potential consumer dissatisfaction [27,28].

Choudhery et al. (1971) have linked sweet curdling with high enzymatic proteolysis that occurs at the exponential growth phase of *Bacillus* [29]. Specifically, some proteases exhibit rennin-like activity by degrading κ-casein (κ-CN) in milk and forming para-κ-CN, which contributes to curd formation [22,29]. Recent research explores the proteolytic capacity of *B. cereus s.l.* strains in various foods, focusing on the genetic basis of psychrotolerance, which enhances the strains’ spoilage capabilities at low temperatures [20,30,31,32]. High-throughput genomic sequencing has significantly advanced our understanding of the genetic make-up of *B. cereus s.l.* strains by enabling the genome-wide identification of key genes associated with resilience, pathogenicity, and spoilage-associated traits [33,34]. These studies have provided valuable insights into the enzymatic and physiological characteristics of spoilage-associated strains, yet the specific molecular mechanisms enabling sweet curdling remain poorly characterized. To this end, this study aims to investigate the genetic basis of *B. cereus*-induced sweet curdling in milk by conducting whole-genome sequencing (WGS) and transcriptome sequencing on a newly isolated strain associated with this phenomenon. The integration of genomic and transcriptomic approaches aims to provide a robust framework for understanding the molecular mechanisms that facilitate *B. cereus* survival and spoilage activity in dairy environments and identify potential genes associated with this process. The insights gained from this work are anticipated to contribute to targeted interventions and strategies in the dairy industry to mitigate spoilage risks.

## 2. Materials and Methods

### 2.1. Bacterial Strain Selection

*B. cereus* strain BC46 (hereafter referred to as “BC46”) was isolated from pasteurized milk from a Greek dairy industry (MEVGAL S.A., Koufalia, Thessaloniki, Greece), as part of a surveillance effort targeting *B. cereus s.l.* strains with potential pathogenic characteristics and spoilage activity in dairy products. For strain isolation, pasteurized cow milk was incubated at 30 °C for 18 h (MIR-250 Cooled Incubator, PHC Europe, Breda, The Netherlands) to promote bacterial growth. The strain was subsequently isolated using COMPASS^®^ Bacillus Cereus Agar (Biokar Diagnostics S.A., Allonne, France).

### 2.2. DNA Extraction and Sequencing of the 16S rRNA Gene

The strain was cultured in sterile Tryptic Soy Broth (TSB) medium (LAB M, Heywood, UK) for 24 h at 30 °C. Subsequently, cells were harvested after centrifugation at 7000× *g* for 10 min. For DNA extraction, we used the Quick-DNA HMW MagBead Kit (Zymo Research, Irvine, CA, USA), following the manufacturer’s instructions. DNA purity was determined using a Nanodrop 2000 spectrophotometer (Thermo Fisher Scientific, Waltham, MA, USA), and DNA was quantified using a Qubit 4.0 Fluorometer with the Qubit™ dsDNA BR Assay Kit (Thermo Fisher Scientific, Waltham, MA, USA). Electrophoresis was performed on a 0.75% *w*/*v* agarose gel in 1× TAE (Tris base, Acetic acid, EDTA) running buffer, stained with ethidium bromide at a final concentration of 0.5 µg/mL, to assess the quality and size of the isolated DNA molecules. The whole 16S rRNA gene sequence (∼1550 bp) was amplified using primers 27F (5′-AGA GTT TGA TCM TGG CTC AG-3′) and 1518R (5′-AAG GAG GTG ATC CAN CCR CA-3′). A Polymerase Chain Reaction (PCR) was performed with the 2X KAPA Taq ready mix (KAPA BIOSYSTEMS, Woburn, MA, USA) under the following conditions: 95 °C for 3 min, 35 cycles of 95 °C for 30 s, 58 °C for 30 s, and 72 °C for 90 s, followed by a final extension at 72 °C for 10 min. PCR products were purified using AMPure XP beads (Beckman Coulter, High Wycombe, UK). Sanger sequencing was performed using the BigDye™ Terminator 3.1 method and sequencing reactions were purified using ethanol/EDTA precipitation. Capillary electrophoresis was conducted on an ABI 3500 Genetic Analyzer (Thermo Fisher Scientific, Waltham, MA, USA). Sequences were analyzed using the BioEdit Sequence Alignment Editor (https://thalljiscience.github.io/, accessed on 20 February 2025) and taxonomy classification was performed using the Basic Local Alignment Search Tool (BLAST, https://blast.ncbi.nlm.nih.gov/Blast.cgi, accessed on 20 February 2025).

### 2.3. Whole Genome Sequencing

#### 2.3.1. Short-Reads Sequencing

For the construction of the genomic library of short-read sequences we employed the Illumina^®^ DNA Prep kit (Illumina Inc., San Diego, CA, USA) following the manufacturer’s instructions. AMPure XP paramagnetic nucleic acid binding beads (Beckman Coulter, Brea, CA, USA) were used to clean the libraries of unbound nucleotides, excess primers, and primer dimers. Library size and quality were assessed with the 5200 Fragment Analyzer system (Agilent Technologies Inc., Santa Clara, CA, USA) using the DNF-915-K0500 kit. The KAPA Library Quantification kit for Illumina sequencing platforms (KAPA BIOSYSTEMS, Wilmington, MA, USA) was used for quantification. Libraries were sequenced on a MiSeq platform (Illumina Inc., San Diego, CA, USA) with the MiSeq^®^ reagent kit v3 (600 cycles).

#### 2.3.2. Long-Reads Sequencing

For long-read Nanopore sequencing, we used 1 μg of high molecular weight DNA to carry out the Native Barcoding Expansion protocol in conjunction with the Ligation Sequencing Kit (EXP-NBD104, SQK-LSK109; Oxford Nanopore Technologies, Oxford, UK). The NEBNext^®^ FFPE DNA Repair, Ultra™ II End Repair/dA-Tailing and Quick Ligation Modules (New England Biolabs Inc., Ipswich, MA, USA) were used for DNA repair, end-prep, and barcode ligation, respectively. Library clean-up steps were performed using AMPure XP beads (Beckman Coulter, Brea, CA, USA). The adapter ligated library was purified using the Short Fragment Buffer (SFB) to retain DNA fragments of all sizes and was quantified using the Qubit™ 1× dsDNA High Sensitivity Assay Kit (Thermo Fisher Scientific, Waltham, MA, USA). Finally, the prepared library was loaded on an R9.4.1 (FLO-MIN106) flowcell and was sequenced on a MinION Mk1B device using the MinKNOW v23.04.3 software (Oxford Nanopore Technologies, Oxford, UK).

#### 2.3.3. Genome Assembly and Annotation

The short reads’ quality was evaluated using FastQC v0.11.7 [35]. Trim Galore! v0.6.7 [36] was used with default parameters for filtering sequencing adapters, unidentified nucleotides (Ns), and low-quality read ends (minimum Q-score 28). Long reads were processed for base calling and demultiplexing in real-time using the fast model of Guppy v6.5.7+ca6d6afb8, with a minimum Q score threshold of 8 and a minimum read length of 200 base pairs (bp), and were subsequently summarized using pycoQC v2.5.2 [37]. Long reads were filtered and trimmed of adaptor sequences using PoreChop ABI v0.5.0 [38], and basic sequencing statistics were generated with NanoPlot v1.42.0 [39]. The genome assembly was constructed from long reads with the Trycycler pipeline v0.5.5 [40]. Briefly, after the removal of the 5% lowest quality reads using Filtlong v0.2.1 (https://github.com/rrwick/Filtlong, accessed on 20 February 2025), the reads were randomly subsampled across 12 iterations. Flye v2.9.4-b1799 [41], Miniasm/Minipolish v0.3-r179/v0.1.3 [42], and Raven v1.8.3 [43] were each used to generate 4 assemblies from the 12 independent read subsets. We, then, examined the assembly graphs in Bandage v0.8.1 [44], and excluded highly fragmented assemblies from further analysis. The Trycycler commands for clustering, reconciling, multiple sequence alignment (MSA), partitioning, and consensus generation produced a refined consensus assembly. The assembled genome was long-read polished with Medaka v1.11.1 (https://github.com/nanoporetech/medaka, accessed on 20 February 2025) and then short-read polished using Polypolish v0.6.0 [45] and Pypolca v0.3.1 [46,47]. The assembled genome’s quality was assessed using QUAST v5.0.2 [48] and its completeness with CheckM v1.2.3 [49]. Putative genomic islands (GIs) were predicted using the Genomic Island Prediction Software (GIPSy) v.1.1.3 program [50]. The inclusion criteria for inferred GIs included a sequence length exceeding 9–10 Kb and a GIPSy prediction score of “Normal” or “Strong”. Plasmid detection was performed with blastn against the PLSDB database v.2024_05_31_v2 [51], and was further confirmed using the PLASMe v1.1 tool [52]. Genome annotation was performed with Bakta v1.9.4 [53] with the parameter “--genus” set to “Bacillus”. The eggNOG-mapper v2 webserver [54] was used to assign protein sequences to Clusters of Orthologous Genes (COG) functional categories in the eggNOG v5.0 database [55,56]. The presence of antimicrobial resistance (AMR) genes was predicted using the NCBI AMRFinderPlus v3.12.8 [57], ResFinder v4.6.0 [58], and Resistance Gene Identifier (RGI) v6.0.3 [59] tools. Virulence and toxin genes were detected using BTyper3 v3.4.0 [60]. The circular genome map of strain BC46 was generated within the Proksee web server (https://proksee.ca/, accessed on 20 February 2025) [61].

#### 2.3.4. In Silico Species Identification and Typing

The taxonomic assignment of strain BC46 was performed in silico using the 2020 standardized genomospecies/subspecies/biovar taxonomy for *B. cereus s.l.* [62] via the BTyper3 v3.4.0 tool [60]. The complete genome sequence of the strain was processed using BTyper3, which utilizes Average Nucleotide Identity (ANI)-based genomospecies assignment, subspecies assignment, and Multi-Locus Sequence Typing (MLST) for in-depth molecular typing. Genome data were submitted to the Type Strain Genome Server (TYGS) for a whole genome-based taxonomic classification by comparing against a comprehensive database for type strains [63]. FASTME 2.1.6.1 was used to construct a midpoint-rooted phylogenetic tree which was visualized with PhyD3 within the TYGS web server [64,65,66].

### 2.4. RNA Sequencing and Differential Gene Expression (DGE) Analysis

#### 2.4.1. *Bacillus* Spore Formation and Milk Inoculation

To study the changes in the gene expression profile of strain BC46, we inoculated ultra-high temperature (UHT)-treated milk samples with bacterial endospores and incubated them to facilitate bacterial growth, and, thus, sweet curdling in milk. Specifically, for inoculum preparation, bacterial cultures (Brain Heart Infusion medium—BHI, Neogen, Lansing, MI, USA) were transferred onto a sporulation agar containing 5 g/L peptone (Neogen, Lansing, MI, USA), 3 g/L yeast extract (LAB M, Heywood, UK), 0.5 mM MnSO_4_·H_2_O (Penta s.r.o., Prague, Czech Republic), and 15 g/L agar (Neogen, Lansing, MI, USA). The plates were incubated at 30 °C for 4–5 days or until more than 80% endospores were observed as determined by phase contrast microscopy. For the collection of the heat-resistant spores, the agar plate cultures were washed and gently scraped with 5 mL sterile dH_2_O and a glass rod. Spore suspensions were heat-shocked in a water bath at 80 °C for 10 min to eliminate vegetative cells and afterwards immediately cooled on ice. The heat-shocked cultures were washed 3 times with sterile Phosphate Buffer Saline (PBS) by centrifugation at 4000× *g* for 20 min, at 4 °C (Sorvall RC 26 Plus, Kendro Lab., Bad Homburg, Germany). To determine the initial population, harvested spores were plated onto Tryptic Soy Agar—TSA (LAB M, Heywood, UK) and incubated for 24 h at 30 °C. For the investigation of the BC46 expression profile before and after sweet curdling of milk, UHT milk samples (0.5 L) were inoculated with 100 CFU/mL spore suspension and incubated at 30 °C for 18 h. The experiment included three replicates (3 milk samples of 0.5 L each), as well as a negative control sample which comprised 0.5 L of UHT milk of the same batch, held under identical conditions but without addition of the bacterial strain, to verify that no undesirable bacterial growth occurred during the experiment.

#### 2.4.2. Strain BC46 Growth and Defect Monitoring in Milk

To monitor BC46 growth in UHT milk at 30 °C after endospore inoculation, samples were collected every 2 h and analyzed via the pour plate method on Tryptone Soya Yeast Extract Agar (LAB M, Heywood, UK). Sweet curdling was identified through the visual observation of protein coagulation at the bottom of the transparent glass containers, and pH measurements were concurrently conducted to verify that no acidification occurred during the defect. Throughout the experiment, we regularly collected samples from the negative control milk and assessed bacterial contamination using the pour plate method on Tryptone Soya Yeast Extract Agar, confirming the absence of bacterial growth.

#### 2.4.3. Sample Collection

We collected milk samples at two distinct time points: during the exponential growth phase, before any defect was observed, and during the stationary phase, after sweet curdling occurred. The sample codes (EA, EB, EC, SA, SB, SC) represent both the growth phase (E for exponential, S for stationary) and the biological replicate (A, B, C). Accordingly, group T1 (7 h of incubation) samples include EA, EB, and EC, while group T2 (15 h of incubation) samples comprise SA, SB, and SC. To obtain high-quality, pure bacterial RNA, the samples were treated with sterile 50% trisodium citrate solution (C_6_H_5_Na_3_O_7_·2H_2_O; Merck KGaA, Darmstadt, Germany) in a 1:25 ratio. Sodium citrate served as a buffer and emulsifying salt to facilitate effective bacterial cell release for collection [67,68]. Samples were centrifuged at 4000× *g* for 20 min at 4 °C (Sorvall RC 26 Plus, Kendro Lab., Bad Homburg, Germany), and the bacterial pellet was resuspended in DNA/RNA Shield™ solution (Zymo Research Inc., Irvine, CA, USA) and kept at −80 °C until RNA extraction.

#### 2.4.4. RNA Extraction and Sequencing

RNA extraction was performed with the ZymoBIOMICS™ RNA Miniprep kit (R2001, Zymo Research Inc., Irvine, CA, USA) according to the manufacturer’s instructions. The RNA concentration was determined using the Qubit 4.0 Fluorometer with the Qubit^®^ RNA BR assay kit (Thermo Fisher Scientific, Waltham, MA, USA). The quality and integrity of RNA molecules were determined on the 5200 Fragment Analyzer system using the DNF-471-0500 kit (Agilent Technologies Inc., Santa Clara, CA, USA). The RNA libraries were constructed with the NEBNext^®^ Ultra™ II RNA Library Prep Kit for Illumina^®^ (E7770, New England Biolabs Inc., Ipswich, MA, USA) according to the manufacturer’s instructions, after the removal of ribosomal RNA (rRNA) with the NEBNext^®^ rRNA Depletion Kit (Bacteria). AMPure XP beads (Beckman Coulter, Brea, CA, USA) were used to clean the libraries from free nucleotides and excess primers. The library size and quality were assessed with the 5200 Fragment Analyzer system using the DNF-474-0500 High Sensitivity NGS Fragment Analysis kit (Agilent Technologies Inc., Santa Clara, CA, USA). The KAPA Library Quantification kit for Illumina sequencing platforms (KAPA BIOSYSTEMS, Wilmington, MA, USA) was used for library quantification. Sequencing was performed on a MiSeq platform (Illumina Inc., San Diego, CA, USA) using the MiSeq^®^ reagent kit v3 (600 cycles).

#### 2.4.5. Bioinformatics and DGE Analysis

Sequence quality assessment was conducted using the FastQC v0.11.7 tool [35]. Sequences with low quality (Q-scores below 30), adapter contamination, unidentified bases (N), and lengths under 18 nucleotides were filtered out using Fastp v0.23.4 [69]. rRNA reads were removed using the SortMeRNA v2.1b program [70]. We aligned the filtered reads to the assembled BC46 genome with the STAR v2.7.11b aligner, using the “--quantMode” parameter set to “GeneCounts” to calculate read counts [71]. R v4.4.1 [72] and the DESeq2 v1.44.0 package [73] were used for differential gene expression (DGE) analysis; read counts were normalized as log2(fold change) (l2fc), and *p*-values were calculated with the Wald test adjusted by the Benjamini–Hochberg method. The “apeglm” estimator was applied to correct for low counts and high dispersion, and genes with an adjusted *p*-value < 0.05 and absolute l2fc > 2 were considered significantly differentially expressed (DEGs). Gene set enrichment analysis was performed against the STRING.1396.Bacillus genome (STRINGv11.5) using the ShinyGO v0.80 web server [74], with significantly enriched Gene Ontology (GO) terms identified at False Discovery Rate (FDR) < 0.05. Figure plots were generated using the R packages *ggplot2* [75], *pheatmap* [76], and *EnhancedVolcano* [77].

## 3. Results and Discussion

### 3.1. Genomic Features and Taxonomy Classification of Strain BC46

**Figure 1:**
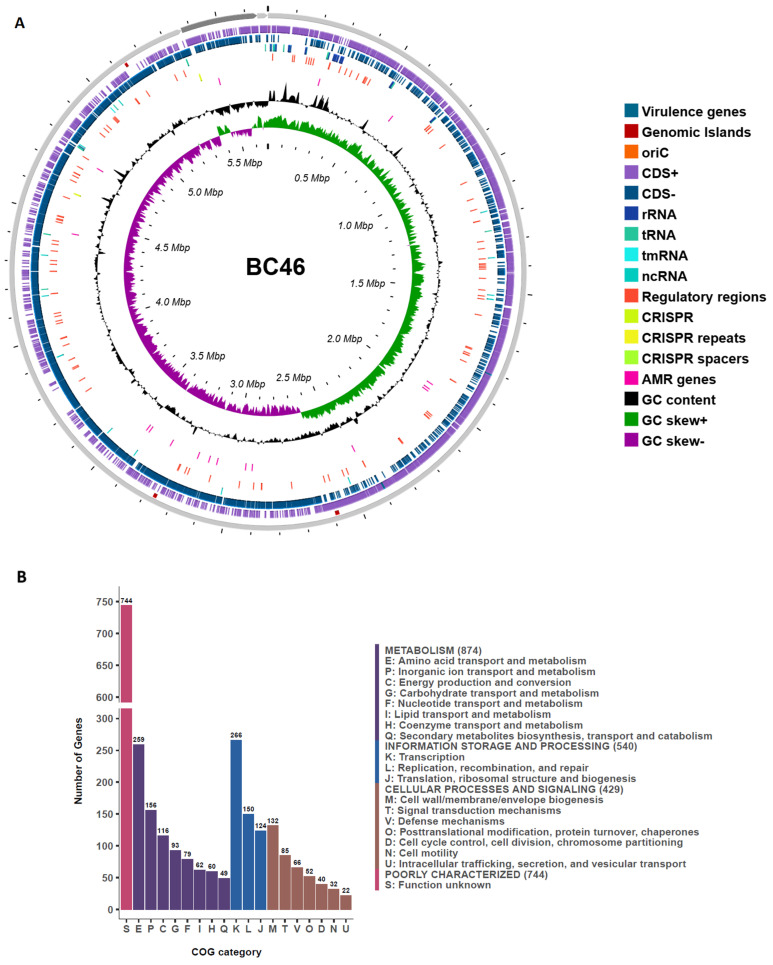
(**A**). Circular map graph of the genomic components and annotation of *B. cereus* strain BC46. From outer to inner circle: contigs (scale ×0.1 Mb), genomic islands (GIs), CDS on the forward strand, CDS on the reverse strand, RNA genes, regulatory regions genes, CRISPR elements, homologous CDS to known antimicrobial resistance (AMR) genes, GC content and GC skew (+/−) calculated as (G−C)/(G+C). (**B**). Clusters of Orthologous Groups (COG) classification of BC46 protein sequences.

The resilience of *B. cereus s.l.* under adverse environmental conditions poses a significant concern for public health, food safety, and product quality. This resilience complicates their elimination from food products, underscoring the importance of efficient monitoring protocols and better understanding of their genetic makeup, to better inform management protocols. Continuous genomic studies of new isolates can yield valuable insights into the resistance and pathogenicity mechanisms that contribute to their persistence [78]. Whole genome characterization of strain BC46 was performed to provide evidence for genetic determinants that support its enhanced survival and spoilage capacities. The hybrid assembly of strain BC46 resulted in three contigs with L50 and N50 values of 1 and 5,370,328, respectively. The genome spans a total length of 5,681,541 bp with a GC% content of 35.1%, similar to those reported previously for *B. cereus* [79]. A summary of the key genome characteristics is presented in Table 1. CheckM analysis further validated the assembly, reporting a near-complete genome at 99.41% and a contamination level of 0.15%. The genome of strain BC46 included 5607 predicted coding sequences (CDS), 18 pseudogenes, 107 transfer RNA (tRNA), 42 ribosomal RNA (rRNA), 1 transfer-messenger RNA (tmRNA), and 17 non-coding RNA (ncRNA) genes. In addition, two Clustered Regularly Interspaced Short Palindromic Repeats (CRISPR) arrays were predicted, with three and five repeats each. The circular genome visualization of strain BC46 is represented in Figure 1A.

Genomic islands (GIs) are discrete DNA segments that harbor gene sets contributing to adaptability, pathogenicity, and ecological success in bacterial strains [80]. They are widely regarded as regions of probable horizontal gene transfer. The GIPSy tool employs a series of analytical steps to predict GIs, and subsequently categorizes them based on their functional roles, including Pathogenicity Islands (PAIs), Resistance Islands (RIs), Metabolic Islands (MIs), and Symbiotic Islands (SIs). Three potential GIs were detected in the BC46 chromosome, with sizes ranging from 9.8 Κb to 14.8 Κb (Figure 1A). Four additional predicted GIs, spanning 19.9 Κb to 29.9 Κb, were identified; however, their weak prediction scores necessitate further validation using other GI prediction tools (Table S1). The GC% content is a strong indicator of GI presence within a genome, as it often differs significantly compared to that of the core genome sequence [81]. In the BC46 genome, Putative GI 1 (9.8 Κb) had a 66% GC deviation, while Putative MI 1 (13.2 Κb) and Putative GI 2 (14.8 Κb) deviated by 16% and 35%, respectively. These GIs consisted of a total of 38 genes associated with capsule biosynthesis, cell wall integrity, utilization and transport of substrates, and sporulation proteins (Table S2). This suggests that BC46 acquired some adaptability and virulence mechanisms by horizontal gene transfer, as reported previously for *B. cereus s.l.* strains [82]. Additionally, the comparison of two smaller circular contigs with the PLSDB and PLASMe databases indicated the potential presence of plasmids within the genome of strain BC46. Contig #2 (273.3 Κb) showed the highest similarity to the *B. cereus* strain JHU plasmid p2 (CP046513.1), whereas contig #3 (37.8 Κb) aligned most closely with the *B. cereus* FRI-35 plasmid p03 (NC_018499.1). The presence of *cry* genes encoding for crystalline entomocidal protoxins in the putative plasmid (contig #2) of strain BC46 is another indicator of possible horizontal gene transfer, probably from *B. thuringiensis* strains, which typically possess insecticidal properties [83]. Gene distribution events in natural environments, such as farm soil and feed rations, are observed between closely related *B. cereus s.l.* isolates [84]. In contig #2, a CRISPR array containing five repeats was identified along with multiple *cas* genes (*cas3*, *cas5c*, *cas8c*, *cas7c*, and *cas2*), and phage proteins, which indicates an acquired adaptive defense mechanism in BC46. The strain’s plasticity was also confirmed by transposases present in contig #2, enhancing its adaptability capacity in competitive and nutrient rich environments, such as milk.

A total of 3001 protein sequences were functionally annotated through the eggNOG-mapper, with 91.8% (2587) mapped to the COG database (Figure 1B). Among them, metabolism-related functions comprised 33.7% of the annotations, with the majority related to amino acid transport and metabolism (259 genes), inorganic ion transport and metabolism (156 genes), and energy production and conversion (116 genes). This indicates the strain’s metabolic flexibility enabling adaptability to fluctuating nutrient levels and environmental conditions. Furthermore, 20.9% of these genes were associated with functions of information, storage, and processing, including 266 genes related to transcription. This highlights the strain’s potential for gene expression regulation, which is crucial for adapting to environmental changes. These results highlight the strain’s enhanced potential for survival and competitiveness across the dairy production line. Cellular processes and signaling comprised 16.6%, primarily cell wall/membrane/envelope biogenesis (132 genes), which could be implicated in resilience against antimicrobial agents or response to external stimuli. Additionally, 744 genes were categorized under unknown functions. While a portion of these may represent novel or poorly characterized proteins, the presence of such a significant number of unknown functions suggests that further investigations are necessary to elucidate their roles. A similar classification pattern of protein functions was observed in *B. cereus* strain 121, isolated from pasteurized milk, except for the absence of cell motility genes [85]. *B. licheniformis* strain 919, isolated from powdered infant formula, exhibited a slightly different distribution of COG categories, with a higher representation of genes associated with carbohydrate transport and metabolism (G) as well as posttranslational processes (O) compared to our strain [85]. These variations in gene distributions suggest that dairy spoilage *Bacillus* strains exhibit metabolic and/or environmental adaptations reflecting their ability to adjust to different environmental conditions.

**Figure 2:**
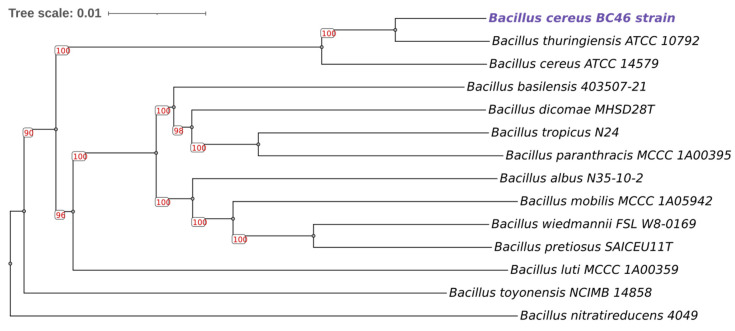
Genome BLAST Distance Phylogeny (GBDP) tree based on whole genome sequences. Branch lengths reflect GBDP distances, and pseudo-bootstrap support values above 60% from 100 replicates are indicated. The average branch support is 98.5%. The tree comprises *B. cereus* strain BC46 (purple color) alongside 13 *Bacillus* type strains.

16S rRNA sequencing identified the strain BC46 at the group level (Supplementary File S4) consistent with previous findings regarding closely related species within the *B. cereus* group. Species-level identification was achieved through whole-genome sequencing (WGS). According to the eight-group *panC* typing method, the strain was categorized to group IV and the *Bacillus cereus sensu stricto* species. The BTyper3 analysis further supported the *B. cereus s.s.* classification with an Average Nucleotide Identity (ANI) of 96.34%, while no subspecies was detected. The closest type strains were *B. thuringiensis* and *B. cereus*, with an ANI of 98.08% and 96.87%, respectively. The phylogenetic analysis using the Type Strain Genome Server (TYGS) confirmed that BC46 belongs to *B. cereus s.s.* and is clustered along with the *B. thuringiensis* type strain ATCC 10792 and the *B. cereus* type strain ATCC 14579 in a unique branch (Figure 2). Additionally, the PubMLST seven-gene multilocus sequence typing (MLST) method indicated a sequence type (ST) of 1009 with no associated clonal complex, based on perfect matches. The absence of a clonal complex associated with ST 1009 might suggest the presence of unique metabolic characteristics that differentiate strain BC46 from other common lineages. This highlights the importance of genomic studies into novel *B. cereus s.l.* isolates from dairy sources, to map the genetic determinants of spoilage.

### 3.2. Identification of Virulence Factors, Antimicrobial, and Cold Resistance Genes

Some *B. cereus s.l.* strains are emerging pathogens with intrinsic antibiotic resistance or toxin production, in addition to their spoilage capacity. *B. cereus s.s.* is known for producing various toxins that contribute to foodborne illnesses. The BC46 genome contains a broad repertoire of genes encoding for enterotoxins (Figure 1A). Specifically, *nheA*, *nheB*, and *nheC* genes, which are components of the non-haemolytic enterotoxin (Nhe) were identified, along with the *hblABCD* gene cluster responsible for haemolysin BL (Hbl) toxin production. The BC46 chromosome also contains cytotoxin K and sphingomyelinase encoding genes, *cytK2* and *sph*, respectively. The above toxins are associated with severe food poisoning cases and gastrointestinal diarrheal disease [2,86]. Notably, two genes encoding for enterotoxin family proteins were identified in contig #2. Furthermore, the putative enterotoxin genes *entA* and *entC*, along with *entFM*, were identified, as were genes encoding metalloprotease immune inhibitors (*inhA1* and *inhA2*) and *camelysin*. Lastly, genes involved in motility, such as flagellar proteins (*fliF*, *motB*, and *flagellins*) and chemotaxis-related proteins (*cheA* and *cheY*) were detected in the core genome, while flagellin genes were also detected in contig #2 of strain BC46 (Table S2). These proteins take part in the flagellum assembly and function, and their presence suggests an enhanced motility capacity of strain BC46, which is crucial for bacterial colonization and infection of the host [87].

In the context of cold-stored food products, such as milk and dairy, identifying the cold shock response characteristics of bacterial contaminants is of utmost importance. Mesophilic and psychrotolerant *B. cereus s.l.* are able to withstand fluctuations in temperature [7]. BC46 contains cold shock protein (Csp) genes, which play a crucial role in the ability to adapt and grow at low temperatures [88]. Specifically, we identified the *cspC* and *cspD* genes, along with two *cspB*-like genes, one *cspE*-like gene, and two putative *csp* genes (Table S2). In addition, the *cspA* gene encoding an RNA chaperone/antiterminator CspA, along with its ncRNA region of thermoregulator were also found. Other identified genes implicated in psychrotolerance were the DEAD-box helicases genes *cshA*, *cshB*, *cshC*, and *cshE*, and chaperone *dnaJ*. This finding aligns with previous studies on the *Bacillus* genus, which associate the presence of *cshA* and *cshB* genes with the cold-resistance phenotype of *B. subtilis* [89]. Porcellato et al. (2021) showed that psychrotrophic *B. cereus s.l.* isolates from milk carried enterotoxins genes [90] which is also consistent with our findings. These results further implicate the role of strain BC46 in sweet curdling of milk, since the psychrotolerance-related genes could enable even vegetative cells to persist during milk cold-storage, consequently leading to defective batches and product loss.

The spread of antimicrobial resistance (AMR) is recognized as one of the most pressing health challenges globally [91]. AMR genes associated with resistance to one or more antimicrobials were predicted in the genome of strain BC46. *B. cereus s.l.* strains are predominantly resistant to beta-lactam antibiotics, such as penicillin, ampicillin, and cefotaxime [92]. A total of 12 AMR-related genes were identified in BC46. Among them were a *fosB* gene encoding a fosfomycin resistance protein, beta-lactam resistance genes (*bla*, *bla2*), vancomycin and teicoplanin resistance genes (*yoaR*, *ldcB*, *vanY*, *vanW*), and *satA* gene related to streptothricin resistance (Table S3). In contig #3, among the 40 genes identified, 13 were lantibiotic-related, which are proteins that exhibit potent activity against Gram-positive bacteria [93]. These genes potentially contribute to the competitiveness of BC46 in diverse microbial environments through acquired antimicrobial mechanisms. However, given the strain’s high potential for horizontal gene transfer, these findings raise significant public health implications regarding AMR spread through the dairy food chain, highlighting the need for targeted efforts for early detection in the dairy processing environment.

### 3.3. Differential Gene Expression Analysis of Strain BC46 During Sweet Curdling of Milk

The pH of fresh, unspoiled cow milk is typically between 6.5 and 6.7, while lower pH values indicate bacterial deterioration [94]. The fermentation of lactose by lactic acid bacteria decreases the pH to 3.9–4.4 in UHT milk [95]. The BC46 strain’s growth in UHT milk at 30 °C indicated that sweet curdling began to manifest after 13 h of incubation and was fully established by 15 h, without a significant drop in pH (Table 2). To further investigate the mechanisms related to sweet curdling, we performed RNA sequencing of strain BC46 to identify the genes expressed before (T1; 7 h of incubation) and after (T2; 15 h of incubation) milk coagulation. In T1 samples, uniquely mapped reads on the BC46 genome accounted for 96.92–97.63% of the total reads, while in T2 samples, they accounted for 98.11–98.57%. The obtained number of raw reads, the remaining reads after quality filtering, and the percentage of mapped reads to the BC46 genome are presented in Table S4.

**Figure 3:**
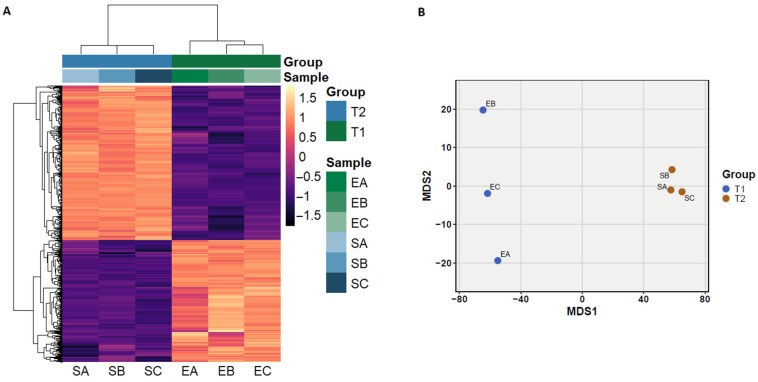
Differential gene expression (DGE) analysis of the *B. cereus* strain BC46 before and after sweet curdling. (**A**). Heatmap of DEGs of stationary growth phase samples (T2; SA, SB, SC) where the sweet curdling defect was observed versus exponential growth phase samples (T1; EA, EB, EC) where no milk spoilage had occurred. The heatmap depicts all differentially expressed genes. Rows and columns were clustered using Euclidean distances and the “complete” method, and values were centered and scaled in the row direction. The color scale (purple to orange) represents rlog-transformed DEGs. (**B**). Multidimensional scaling (MDS) plot for all samples using the Euclidean distances of rlog-transformed counts.

The mapping of the reads to the BC46 genome yielded 5981 transcripts. Filtering out transcripts with read counts of 0 or 1 across all samples reduced the dataset to 2726 transcripts. Of these, 950 were differentially expressed (absolute l2fc > 2, *p*-adjusted < 0.05). Among them, 531 were upregulated in sweet curdling samples (T2), while 419 were downregulated (Figure 3A). This indicates dynamic changes in transcript expression associated with the transition between growth phases, potentially influencing the curdling process. Multidimensional scaling (MDS) based on the gene expression profile of the samples further supports this finding, revealing two separate clusters, that suggest significant differences in expression patterns associated with the growth phase and curdling process (Figure 3B). Furthermore, functional enrichment analysis classified genes into biological process, cellular component, and molecular function GO terms. Overall, 103 unique biological process terms (GO-BP), 10 cellular component terms (GO-CC), and 9 molecular function terms (GO-MF) were enriched at FDR < 0.05. ShinyGO could not successfully annotate all the DEGs; thus, the results were based on a lower number of genes. We observed major differences in the enriched pathways between the T1 and T2 samples. Key pathways in exponential phase (T1) included intracellular organelle and ribosome components (GO-CC), RNA binding and ligase activities (GO-MF), amino acid salvage (GO-BP), and histidine biosynthesis processes (GO-BP) (Figure S1). These pathways suggest protein and amino acid synthesis during the exponential growth phase where rapid cell division is required. In contrast, upregulated pathways in T2 and sweet curdling were related to energy metabolism, transport mechanisms, and membrane integrity, such as oxidoreductase activity (GO-MF), the glycerol-3-phosphate metabolic process (GO-BP), and the pyrimidine and Uridine monophosphate (UMP) metabolic and biosynthetic processes (GO-BP) (Figure S2). The upregulation of oxidoreductases genes, such as *nuoN* and *yrpB*, indicating the overexpression of complex I of the electron transport chain in our findings, aligns with previous studies on *B. cereus* biofilms [96]. These distinct patterns indicate the strain’s adaptability to environmental changes, which are crucial for its survival and spoilage capacity in dairy food products.

**Figure 4:**
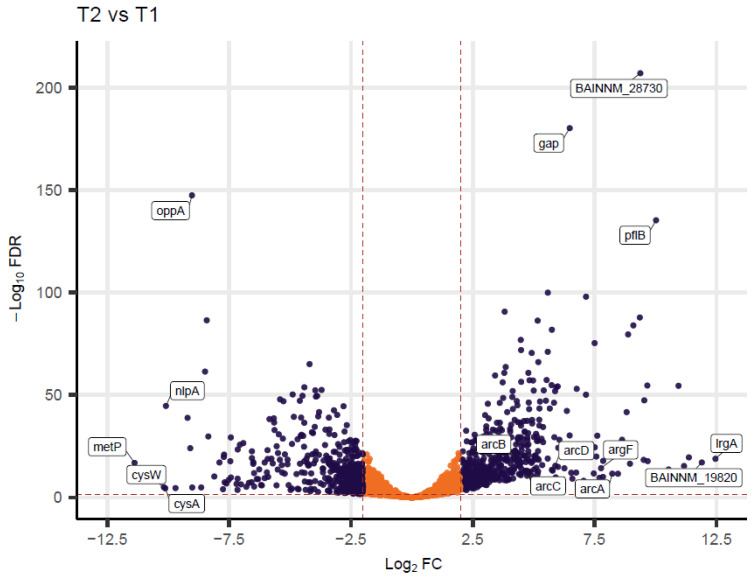
Volcano plot depicting differentially expressed genes (DEGs) in T2 versus T1 samples. Purple dots represent DEGs with *p*-adjusted < 0.05 and absolute l2fc > 2. Y-axis denotes − log10 *p*-adjusted values while X-axis shows log2(fold change) values. The top 5 up- and downregulated genes with an adjusted *p*-value < 0.05 and absolute l2fc > 2, along with the genes involved in the ADI pathway, are highlighted.

The differentially expressed genes (DEGs) in T2 versus T1 samples are illustrated in the volcano plot shown in Figure 4. Highly expressed genes in T1 samples were involved in cellular nutrition (*oppA*), amino acid biosynthesis (*cysA*, *cysW*), and transport (*nlpA*, *metP*), which are linked to the strain’s exponential growth phase [97,98]. The top highly expressed genes in T2 samples included *lrgA*, *pflB*, *gap*, a putative phosphoesterase gene (BAINNM_19820), and an uncharacterized protein (BAINNM_28730). Previous studies have demonstrated that glyceraldehyde-3-phosphate dehydrogenase (GAPDH), encoded by the *gap* gene, plays a crucial role in *B. cereus* biofilm formation by controlling the expression of *lrgA* and *lrgB* genes [99]. The overexpression of these genes, along with *lrgB*, during BC46-induced sweet curdling suggests their potential involvement in biofilm formation during this phenomenon. The genes *pflA* and *pflB* were upregulated in T2 samples. These genes have been implicated in the oxidative stress response in *Staphylococcus aureus* and have been shown to be crucial for the survival of its biofilms [100]. This suggests that the highly expressed genes in strain BC46 during sweet curdling contribute to a robust adaptive mechanism, which may facilitate its persistence in various environmental conditions, including dairy processing environments. Among the highly expressed genes upregulated in T2 samples, several were found to be involved in biological processes, including the glycerol-3-phosphate metabolic process, the pyrimidine ribonucleoside monophosphate metabolic process, the pyrimidine ribonucleotide metabolic and biosynthetic process, and oxidoreductase activity (Figure S2). Of them, *accC* (acetyl/propionyl-CoA carboxylase, alpha subunit), *aspA* (aspartate ammonia-lyase), and *ndk* (nucleoside-diphosphate kinase) have previously been associated with the production of spoilage enzymes in UHT milk caused by *Pseudomonas azotoformans* [101]. This finding suggests that certain genes associated with dairy spoilage are conserved across multiple bacterial species, highlighting a genetic link among dairy spoilage microorganisms. These genes hold potential as genetic markers for spoilage monitoring, contributing to the detection and investigation of diverse bacterial contaminants in the dairy industry.

### 3.4. Gene Expression Alterations During BC46 Growth and Sweet Curdling in Milk

#### 3.4.1. Sporulation and Biofilm Formation

Among the upregulated genes in the T2 group, where sweet curdling had occurred, several genes associated with endospore formation and sporulation were identified, such as *spo0E*, *spoIIAB*, *spoIIAA*, *spoVG*, and *spoVS* (Table S5). These include essential regulatory elements, structural components of the spore coat, and proteins critical for various stages of the sporulation process. Environmental and nutritional signals trigger sporulation in *B. cereus s.l.* cell populations, typically during the stationary phase [102]. Genes such as *spoIIAA*, *spoIIAB*, *spoVG*, and *spoVS* encode products that take part in the regulation of the successful sporulation. Specifically, anti-sigma factor SpoIIAB and anti-anti-sigma factor SpoIIAA regulate the sigma factor SigmaF, which activates the transcription of genes involved in the early stages of endospore formation and which are essential for the engulfment stage [103]. In *B. anthracis* and *B. subtilis*, genes involved in sporulation initiation, regulation, and sigma factor control, such as *sigF*, *spoVG*, and *spoIIAA*, were highly expressed during the transition to sporulation and within the first hour of the process [104,105]. Furthermore, *sigF* expression has been shown to increase during the stationary phase and persist throughout sporulation in *B. cereus* ATCC 14579, aligning with our observations in strain BC46 [106]. Notably, *spoVT* encoding for the late-stage sporulation regulator was downregulated in T2 samples. This finding suggests that some cell populations of strain BC46 exhibit temporal variation in sporulation processes during the BC46 growth in milk, likely aimed at enhancing the survival and persistence of the bacterium in an adverse environment. Furthermore, biofilm formation-related genes, such as *ablA*, *ablB*, *cbpA*, *comER*, and *spoVG* were overexpressed in T2 group. Sporulation and biofilm formation are interconnected regulatory pathways in *B. cereus*. Specifically, *kinB*, which was also overexpressed in T2, encodes a histidine kinase involved in the phosphorylation of the master response regulator Spo0A, which is essential for the transition from vegetative growth to sporulation [107]. Furthermore, the observed downregulation of genes involved in flagellar biosynthesis and motor switch proteins in T2 samples suggests a transition from planktonic to biofilm cell populations. The study of Wang et al. (2020) demonstrated that flagellar assembly and sporulation, among other factors, are important pathways involved in *B. licheniformis* biofilm formation [108]. The observed overexpression of sporulation genes in our T2 samples, therefore, appears to be connected with biofilm formation, in accordance with these studies. However, further investigation is needed to support the activation of biofilm formation and elucidate the underlying mechanisms driving this process.

#### 3.4.2. Proteolysis and Sweet Curdling

Extracellular proteolytic enzymes produced by *B. cereus s.l.* significantly impact the spoilage of dairy products [20]. *Bacillus* produce a wide range of proteases, resulting in diverse proteolytic activity compared to other dairy spoilage bacteria, such as *Pseudomonas* spp. [109]. Unlike *Bacillus*, which can survive pasteurization through the formation of resilient spores, *Pseudomonas* are eliminated during the process. However, these bacteria remain a significant contributor to dairy spoilage due to their highly thermostable extracellular proteases and lipases, which remain active post-pasteurization [110]. Proteinase-related genes upregulated in T2 group samples included the serine protease encoding gene *aprE*, neutral protease *nprB*, and *bepA* gene encoding for a metalloprotease BepA/YfgC. Furthermore, other hydrolase-related genes with putative esterase, lipase, and phospholipase activities were overexpressed in T2 (BAINNM_10545, *pldB*, *estA*, *ypfH*, and *aes*). Upregulation of these genes could suggest an increased capacity to break down milk proteins, potentially contributing to the sweet curdling defect. Further research into the expression of proteolytic genes potentially associated with the sweet curdling phenomenon could inform better management strategies to control *B. cereus s.l.* in dairy environments. Additionally, several toxin-related genes were overexpressed in T2 samples (*hblB*, *hblC*, *ndk*, and *nprB*) (Table S5). The *nprB* gene produces a neutral protease that breaks down host proteins to promote infection [111], and was found in strains with proteolytic capacity associated with dairy products [112]. The activity of *nprB*-encoded protease could also contribute to the breakdown of milk proteins, leading to sweet curdling.

*B. cereus s.l.* undergo metabolic adaptations that enhance their resistance to adverse conditions. Genes related to lysine metabolism, *ldcA* and *ldcB*, were overexpressed in T2 samples (Table S5). A primary product of lysine decarboxylases (LDCs) is cadaverine, which plays a role in intracellular pH regulation, enhancing the cell’s resilience under acidic stress conditions [113]. Other metabolic adaptations of *B. cereus s.l.* to acidic and anaerobic conditions include the L-arginine catabolism by the arginine deaminase system (ADI pathway). This pathway leads to the production of ammonia, which contributes to a pH increase or the prevention of a pH decrease [114]. The *arcDABC* genes, primary components of the ADI pathway, are organized in an operon or complex, along with the *argF* gene in various *Bacillus* species, such as *B. subtilis* and *B. licheniformis* [115]. The synergistic action of these genes ensures the efficient functioning of arginine metabolism, which is crucial for energy production and pH regulation under anaerobic conditions [116]. Previous studies demonstrated that genes associated with the ADI pathway in *B. cereus* strains were overexpressed under acidic conditions (pH 5.4–5.5) [117,118]. Additionally, Park et al. (2021) reported that the *arcA*, *arcB*, *arcC*, and *argF* genes were downregulated in mesophilic *B. cereus* strain BCG^t^ cultures at low temperatures (10 °C) compared to higher temperatures (30 °C) [30]. Consistent with these findings, our results showed that these genes, along with *arcD*, were overexpressed in T2 samples of strain BC46 at 30 °C (Figure 4). The overexpression of the ADI pathway genes during BC46-induced spoilage, combined with the slight drop in pH from 6.45 to 6.28 at the initial stationary phase, indicates a potential key role of these genes in maintaining pH levels above 6 and reducing acidity during the milk coagulation process, resulting in sweet curdling. These genetic factors are proposed as key targets for further investigation to determine their precise involvement in the sweet curdling phenomenon associated with strain BC46.

## 4. Conclusions

This study presents the first investigation of the genetic basis underlying the sweet curdling defect in milk, through genomic and transcriptomic characterization of the novel *B. cereus* strain BC46 inducing this defect. We sequenced the whole genome of strain BC46, and characterized its pathogenic and spoilage-associated genetic features. Hybrid assembly of short and long reads revealed that strain BC46 harbors genes involved in toxin synthesis, cold-resistance, and proteolysis, highlighting its relevance to dairy product safety and quality. Furthermore, transcriptomic analysis of BC46 grown in milk revealed significant gene expression alterations during the development of sweet curdling in milk. The combined genomic and transcriptomic approach offers a robust and comprehensive framework for identifying key spoilage factors in *B. cereus* as well as common spoilage genes conserved among dairy-associated bacteria. Our findings suggest genetic targets potentially implicated in sweet curdling. Although derived from a single strain, these results lay the groundwork for further research into this spoilage phenomenon. The gene targets identified in this study should be further evaluated for their role in the sweet curdling defect using additional strains exhibiting this spoilage capacity. Understanding the molecular mechanisms of sweet curdling can contribute to the development of effective strategies for monitoring and controlling *B. cereus s.l.* in the dairy production chain, ultimately improving product shelf life and safety.

## Figures and Tables

**Table 1:** Genome characteristics of *B. cereus* strain BC46. The assembled genome size in base pairs (bp), number of contigs and their sizes (bp), the N50 and L50 values, the percent in G and C bases, the depth of coverage, and the percentage of estimated contamination are presented. L50 represents the number of contigs that account for 50% of the total assembly length, and N50 is the length of the shortest contig in this set.

Attribute	Value
Assembled genome size	5,681,541 bp
Number of contigs	3
Contig #1 size	5,370,328 bp
Contig #2 size	273,354 bp
Contig #3 size	37,859 bp
N50	5,370,328 bp
L50	1
% G+C Content	35.1%
Sequence coverage completeness	~467×
% Estimated contamination	0.15%

**Table 2:** pH values and bacterial growth phase of *B. cereus* strain BC46 in UHT milk incubated at 30 °C. T1 and T2 indicate the time-points of sampling for RNA sequencing. A, B, and C depict biological replicates of milk inoculated with strain BC46.

Incubation Time (h)	Growth Phase	Control Milk pH	BC46 Milk A pH	BC46 Milk B pH	BC46 Milk C pH
2.5	Lag	6.54	6.54	6.54	6.53
7 (T1)	Exponential	6.47	6.46	6.45	6.43
13	Initial stationary	6.47	6.28	6.29	6.31
15 (T2)	Stationary	6.47	6.29	6.29	6.28

## Data Availability

Raw whole genome data of both short and long reads have been deposited to the NCBI Sequence Read Archive (SRA) under the BioProject PRJNA1199927 and Biosample accession number SAMN45892545. Raw RNA sequencing data have been deposited under the BioProject PRJNA1200566 and Biosample accession number SAMN45903799.

## Supplementary Materials

The following supporting information can be downloaded at: https://www.mdpi.com/article/10.3390/foods14050780/s1. Supplementary file S1: Table S1. Genomic islands (GIs) prediction results by GIPSy in the genome of *B. cereus* strain BC46. The category (Pathogenicity—PAI, Metabolic—MI, Resistance—RI, Symbiotic—SI) for each putative island is presented, along with genetic metrics, number of genes in the island, their position in the genome, their length, the prediction score, and the % coverage of the coding sequence (CDS) present in the island. Table S3. Antimicrobial resistance (AMR) genes predicted in the genome of *B. cereus* strain BC46, based on AMRFinderPlus, ResFinder, and Resistance Gene Identifier (RGI) tools. RGI analysis was performed using the “Strict” cut-off. Table S4. Read metrics for transcriptome data of the *B. cereus* strain BC46 samples. Figure S1. Enriched pathways in *B. cereus* strain BC46 T1 samples (EA, EB, and EC). The top 10 enriched pathways (FDR < 0.05) in each of the three Gene Ontology (GO) categories: Biological Process (GO-BP), Cellular Component (GO-CC), and Molecular Function (GO-MF) are presented on the y axis, while the total number of genes in each pathway is presented on the x axis. Figure S2. Enriched pathways in *B. cereus* strain BC46 T2 samples (SA, SB, and SC). The top 10 enriched pathways (FDR < 0.05) in each of the three Gene Ontology (GO) categories: Biological Process (GO-BP), Cellular Component (GO-CC), and Molecular Function (GO-MF) are presented on the y axis, while the total number of genes in each pathway is presented on the x axis. Supplementary file S2: Table S2. Annotation of *B. cereus* strain BC46 genome. Supplementary file S3: Table S5. Differentially expressed genes of *B. cereus* strain BC46 T2 (15 h of incubation; sweet curdling) versus T1 (7 h of incubation; no defect in milk) samples, with *p*-adjusted < 0.05 and absolute l2fc > 2. Supplementary file S4. The 16S rRNA gene sequence of *B. cereus* strain BC46.
