# Supplementary material for: Genomic and Transcriptomic Profiling of Bacillus cereus in Milk: Insights into the Sweet Curdling Defect
Source: Foods. 2025 Feb 25;14(5):780. doi: 10.3390/foods14050780 (PMC11899114; doi:10.3390/foods14050780)

Supplementary materials

Supplementary file 1:

Table S1. Genomic islands (GIs) prediction results by GIPSy in the genome of *B. cereus* strain BC46. The category (Pathogenicity – PAI, Metabolic – MI, Resistance – RI, Symbiotic – SI) for each putative island is presented, along with genetic metrics, number of genes in the island, their position in the genome, their length, the prediction score, and the % coverage of the coding sequence (CDS) present in the island.

Putative GI	Category	% G+C Deviation	% Codon Usage Deviation	% Virulence factors	% Hypothetical proteins	No. of genes	Position start	Position end	Length (bp)	Prediction score	% Coverage of CDS
Putative GI 1	PAI, SI	66%	33%	55%	0%	9	5,134,540	5,144,337	9,798	Strong	95.5
Putative MI 1	MI	16%	0%	58%	8%	12	3,256,412	3,269,632	13,221	Normal	70.5
Putative GI 2	RI, SI	35%	0%	23%	11%	17	2,580,107	2,594,933	14,827	Normal	66.9
Putative PAI 1	PAI	3%	17%	31%	13%	29	3,616,910	3,636,904	19,995	Weak	91.2
Putative RI 1	RI	21%	0%	13%	4%	24	3,008,098	3,037,034	28,937	Weak	73.4
Putative MI 2	MI	28%	0%	24%	8%	22	4,360,673	4,385,040	24,368	*NA*	72.8
Putative RI 2	RI	0%	14%	9%	23%	22	1,237,133	1,257,019	19,887	*NA*	63.9

Table S3. Antimicrobial resistance (AMR) genes predicted in the genome of *B. cereus* strain BC46, based on AMRFinderPlus, ResFinder, and Resistance Gene Identifier (RGI) tools. RGI analysis was performed using the "Strict" cut-off.

Contig	Gene name	Antibiotic class	Position in contig	% Identity	% Coverage	Prediction tool
Contig #1	*fosB*	fosfomycin	1,963,242...1,963,658	98.08 -98.55	100.00	AMRFinderPlus & ResFinder & RGI
Contig #1	*bla*	beta-lactam	2,433,047...2,433,973 & 3,114,021...3,114,971	95.10-95.74	99.67	AMRFinderPlus & RGI
Contig #1	*satA*	streptothricin	3,076,317...3,076,874	89.67	98.91	AMRFinderPlus
Contig #1	*bla2*	beta-lactam	3,303,544...3,304,317	90.61-94.55	100.00	AMRFinderPlus & RGI
Contig #1	*alr*	glycopeptide antibiotic	237,494...238,663	32.16	*NA*	RGI
Contig #1	*alr*	glycopeptide antibiotic	1,998,484...1,999,710	35.16	*NA*	RGI
Contig #1	*yoaR*	vancomycin; teicoplanin	610,175...611,437 & 2,912,694...2,913,524 & 3,432,175...3,433,086	33.33-44.72	*NA*	RGI
Contig #1	*ldcB*	vancomycin; teicoplanin	1,987,318...1,988,058 & 3,167,503...3,168,222 & 4,433,415...4,434,194	38.54-40.69	*NA*	RGI
Contig #1	*tet*	tetracycline; doxycycline; minocycline; chlortetracycline; demeclocycline; oxytetracycline	2,942,346...2,944,289	46.54	*NA*	RGI
Contig #1	BAINNM_*17400*	vancomycin; teicoplanin	3,417,935...3,418,678	39.60	*NA*	RGI
Contig #1	*vanY*	vancomycin; teicoplanin	4,752,730...4,753,590	61.32	*NA*	RGI
Contig #2	*vanW*	vancomycin; teicoplanin	85,112...86,407	32.02	*NA*	RGI

Table S4. Read metrics for transcriptome data of the *B. cereus* strain BC46 samples.

Sample ID	Raw paired-end reads	Filtered reads	% Uniquely mapped	Average mapped length (bp)	% mapped to multiple loci	% mapped to too many loci	% Unmapped
**EA**	2,594,911	2,227,836	97.10	306.68	1.39	0.02	1.49
**EB**	3,214,713	2,767,086	96.92	307.24	1.56	0.02	1.50
**EC**	3,096,650	2,750,595	97.63	300.14	1.11	0.03	1.22
**SA**	2,409,555	2,043,922	98.21	300.28	0.37	0.21	1.21
**SB**	2,908,600	2,498,038	98.11	283.04	0.31	0.13	1.43
**SC**	2,616,426	2,349,515	98.57	293.80	0.27	0.01	1.15

Figure S1. Enriched pathways in *B. cereus* strain BC46 T1 samples (EA, EB, and EC). The top 10 enriched pathways (FDR <0.05) in each of the three Gene Ontology (GO) categories: Biological Process (GO-BP), Cellular Component (GO-CC), and Molecular Function (GO-MF) are presented on the y axis, while the total number of genes in each pathway is presented on the x axis.

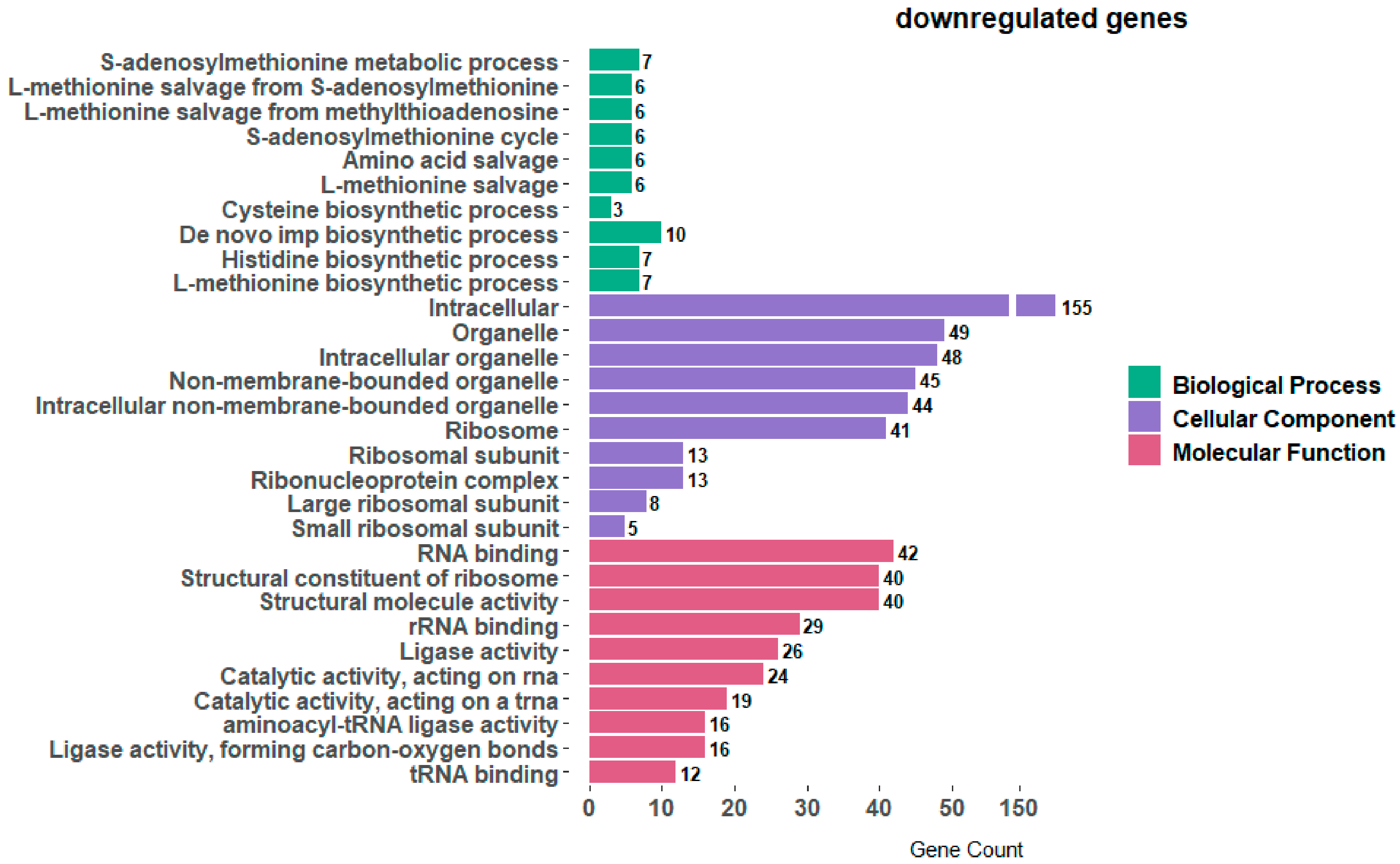

Figure S2. Enriched pathways in *B. cereus* strain BC46 T2 samples (SA, SB, and SC). The top 10 enriched pathways (FDR <0.05) in each of the three Gene Ontology (GO) categories: Biological Process (GO-BP), Cellular Component (GO-CC), and Molecular Function (GO-MF) are presented on the y axis, while the total number of genes in each pathway is presented on the x axis.

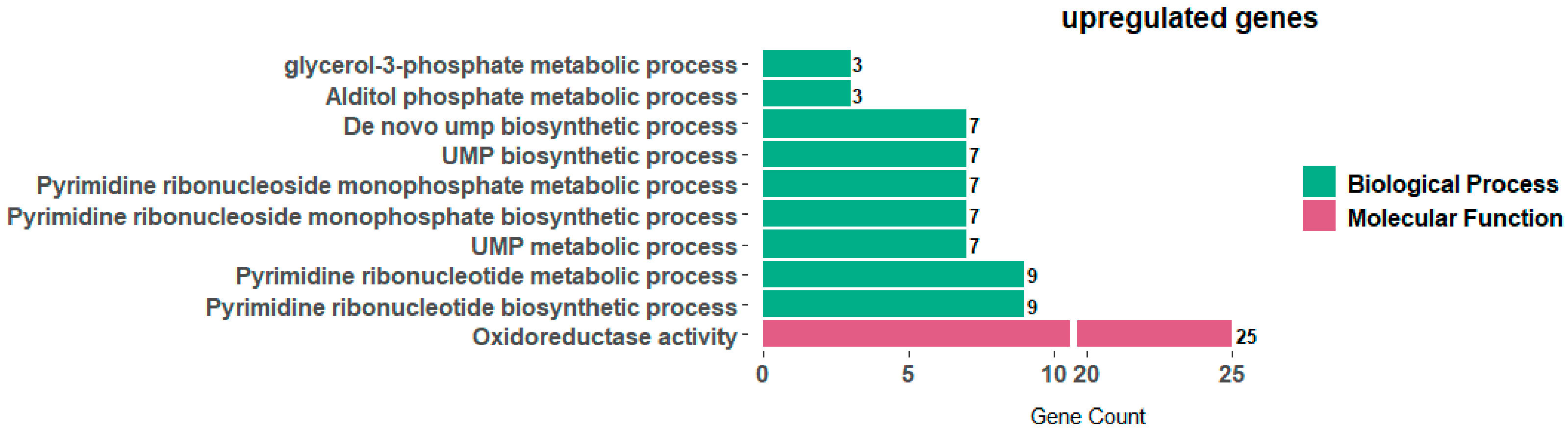

Supplement: Supplementary file 1 [file foods-14-00780-s001.zip › Supplementary_file1.pdf]
